# Efficacy of a slow-release imidacloprid (10%)/flumethrin (4.5%) collar for the prevention of canine leishmaniosis

**DOI:** 10.1186/1756-3305-7-327

**Published:** 2014-07-14

**Authors:** Emanuele Brianti, Gabriella Gaglio, Ettore Napoli, Luigi Falsone, Chiara Prudente, Fabrizio Solari Basano, Maria S Latrofa, Viviana D Tarallo, Filipe Dantas-Torres, Gioia Capelli, Dorothee Stanneck, Salvatore Giannetto, Domenico Otranto

**Affiliations:** 1 Dipartimento di Scienze Veterinarie, Università degli Studi di Messina, Polo Universitario Annunziata, 98168 Messina, Italy; 2 Arcoblu s.r.l., Via Alessandro Milesi 5, 20133 Milano, Italy; 3 Dipartimento di Medicina Veterinaria, Università degli Studi di Bari, Strada Provinciale per Casamassima, 70010 Valenzano, Bari, Italy; 4 Departamento de Imunologia, Centro de Pesquisas Aggeu Magalhães, Recife, Brazil; 5 Istituto Zooprofilattico Sperimentale delle Venezie, Laboratorio di Parassitologia, Legnaro, Italy; 6 Bayer Animal Health GmbH, Leverkusen, Germany

**Keywords:** *Leishmania infantum*, Dog, Canine leishmaniosis, Prevention, Control

## Abstract

**Background:**

The efficacy of a slow-release insecticidal and repellent collar containing 10% imidacloprid and 4.5% flumethrin (Seresto, Bayer Animal Health) in preventing *Leishmania infantum* infection was evaluated in a large population of dogs living in a hyper-endemic area of Sicily (Italy).

**Methods:**

A total of 219 dogs, negative for *L. infantum* were enrolled in a multicentre, controlled study. Dogs were divided into two homogeneous groups, defined as G1 (n = 102) and G2 (n = 117). Before the start of the sand fly season, dogs in G1 were treated with the collar while animals in G2 were left untreated, serving as negative controls. Dogs were serially sampled on day D90, D180, D210 and D300 in order to assess *Leishmania* infection by IFAT, PCR on skin (D210-D300) and bone marrow (D300) and cytology on bone marrow aspirate (D300).

**Results:**

Three dogs (2.9%) in G1 and 41 (40.2%) in G2 became positive for *L. infantum* in at least one of the diagnostic tests employed in the study. The number of seropositive dogs in G2 increased in the course of the study from 15 (D90) to 41 (D300), with some of them also positive in other diagnostic tests. Eight (19.6%) of the seropositive dogs in G2 showed an increase in antibody titers ranging from 1:160 to 1:1,280. At the last follow-up, some of dogs in G2 displayed overt clinical signs suggestive of leishmaniosis. The mean incidence density rate at the final follow-up was 4.0% for G1 and 60.7% for G2, leading to a mean efficacy of the collar in protecting dogs at both sites of 93.4%.

**Conclusions:**

The slow-release collar tested in this study was shown to be safe and highly effective in preventing *L. infantum* infection in a large population of dogs. Protection conferred by a single collar (up to eight months) spanned an entire sand fly season in a hyper-endemic area of southern Italy. The regular use of collars, at least during the sand fly season, may represent a reliable and sustainable strategy for the prevention of leishmaniosis in dogs living in or travelling to an endemic area.

## Background

Canine leishmaniosis (CanL), caused by protozoa of the genus *Leishmania* (Kinetoplastida: Trypanosomatidae), occurs globally and is transmitted through the bites of phlebotomine sand flies (Diptera: Psychodidae) [1]. *Leishmania infantum* is the most important aetiological agent of CanL, and responsible for visceral and cutaneous leishmaniosis in humans in Eurasia and America [2], with an estimated 8.5/100,000 new cases per year in southern European countries (including Turkey) [3,4]. Over the past two decades, the disease has spread northwards from southern Europe [2,5] as a result of several factors such as changes in the distribution of phlebotomine sand fly vectors [6], increased movement of dogs from Mediterranean to central and northern regions [7], and lack of effective control measures [3,8]. Dogs act as reservoir hosts, and the association between CanL and human leishmaniosis has been proven in areas where sand flies are present [1-3]. In particular, dogs with overt clinical signs act as the most important reservoir of *L. infantum* and play a major role in the epidemiology of the infection [9-11]. Therefore, although anti-*Leishmania* treatment of infected dogs reduces clinical signs and parasite load, it does not completely inhibit infection by sand flies [12].

Over the last few decades, several attempts have been made to improve control strategies and to develop reliable and cost-effective preventive measures in dogs [13]. For example, newly developed vaccines have shown efficacy in preventing the progression of active infection and disease [14-17]. Nonetheless, the prevention of sand fly bites through the use of repellent and insecticidal compounds in different formulations (e.g. spot-on, spray, impregnated collars) is regarded as the most effective method of avoiding *L. infantum* infection in dogs [1,10,13] and therefore potentially reducing the risk for human infection [18]. Several pyrethroid-based compounds have been shown to be effective against *Leishmania* infection in dogs living in endemic areas [19-23]. Recently, a polymer matrix collar containing a combination of 10% imidacloprid and 4.5% flumethrin (Seresto®, Bayer Animal Health), referred to in the following as the “collar”, has been licensed for use in dogs and cats [24]. This collar conferred long-term protection (eighth months) against ticks and fleas [25,26] and was successful in preventing the transmission of tick-borne pathogens [27-29]. Although the collar is not currently registered against sand flies, a single preliminary field trial showed its potential for the control of CanL in young dogs from an endemic area [30]. Thus, this study investigates the efficacy of the collar in the prevention of infection by *L. infantum* in dogs of different ages living in two heavily populated shelters located in a hyper-endemic area of the Mediterranean basin.

## Methods

### Ethical statement

A negative-controlled and multicentre study was conducted according to the principles of Good Clinical Practice (VICH GL9 GCP, 2000 http://www.ema.europa.eu/docs/en_GB/document_library/Scientific_guideline/2009/10/WC500004343.pdf) and the guideline on Statistical Principles for Veterinary Clinical Trials (VICH CVMP/816/00, 2000 http://www.ema.europa.eu/docs/en_GB/document_library/Scientific_guideline/2012/01/WC500120834.pdf). The study design and the experimental procedures were approved and authorized by the Italian Ministry of Health (authorization number DGSAF/297-03/04/2012).

### Study sites

**Figure 1:**
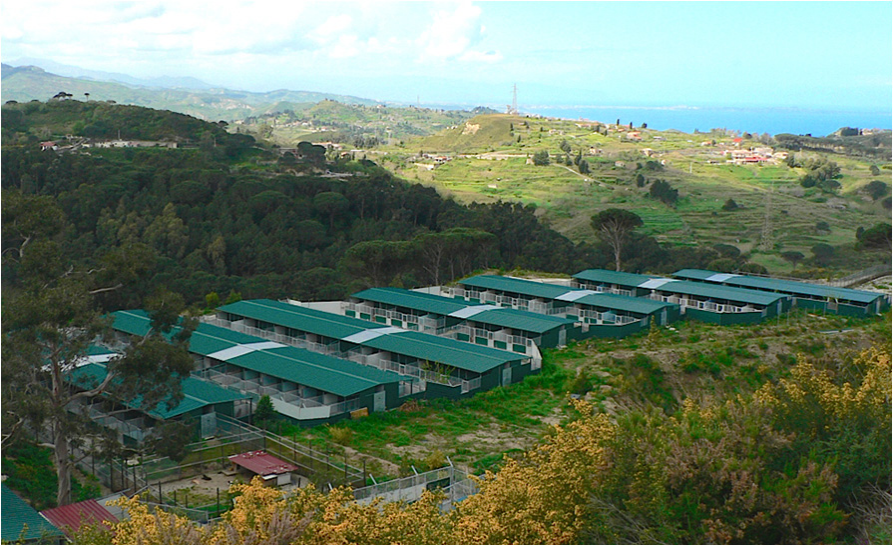
Landscape of the Messina shelter (site S1) in which approximately 450 dogs were housed.

Dogs were housed in two private animal shelters in Sicily (southern Italy), i.e. Messina (N 38.241624°E 15.522016° altitude 520 m), and Augusta (N 37.239034°E 15.135016° altitude 394 m), referred to as site 1 (S1) and site 2 (S2), respectively. The study sites were located in a hyper-endemic area for *L. infantum*[2,5], where the presence of competent sand fly vectors was reported [31,32]. At site S1 approximately 450 dogs were housed in 124 pens measuring 18.4 or 54 m^2^ (Figure 1). Study dogs were housed in pens with a concrete or mixed (concrete and fine gravel) floor, with the pens separated by aluminum composite mesh panels. Site 2 housed approximately 530 dogs in 38 mixed-floor pens each measuring about 100 m^2^. Pens in S2 were separated by iron wire mesh (1×1 cm). In both facilities all the dogs had an adequate covered resting area with dog beds and an open area outside. Dogs in each pen had daily access to wide, shared runs, and were fed with commercial dry dog food and tap water.

### Study design and experimental procedures

In March 2012, before enrolment, a total of 455 dogs (S1 = 144, S2 = 311) were screened for circulating anti-*L. infantum* antibodies using an indirect immunofluorescent antibody test (IFAT) and/or a rapid ELISA test (see below). Only dogs negative at screening were considered as candidates for inclusion in the study. In April-May 2012 (Day 0) these animals were physically examined and weighed, and blood, skin and bone marrow samples were collected (see below). Dogs fulfilling the inclusion criteria (normal general health, ≥ 7 weeks of age, not treated with ectoparasiticidal products or immunosuppressive drugs in the preceding months, and negative for *L. infantum* at serology (IFAT) and in skin and bone marrow PCR and cytology on bone marrow smears from samples collected at the time of inclusion) were enrolled in the study. Included dogs were identified using the microchip code and assigned to one of the two groups, G1 and G2, using a random treatment allocation plan. Randomization was conducted pen-wise instead of allocating single dogs in order to avoid animals from both groups being in physical contact in one pen and active ingredient possibly being transferred from treated to untreated dogs. On the day of inclusion (D0) imidacloprid 10% + flumethrin 4.5% collars were fitted to dogs in G1 on the basis of their body weight (i.e., small collar: < 8 kg/large collar: > 8 kg), while dogs in G2 were left untreated and served as negative controls.

Animals were followed up on days 90, 180, 210 and 300 after inclusion. At each follow-up dogs were physically examined and blood, skin and bone marrow samples were collected as shown in Table 1. The collars were worn from day 0 to 210 and were replaced only if they were lost or if the animal’s weight increased above the threshold of 8 kg. On day 210 the collars were removed from the dogs in group G1 and not replaced. All dogs included in the study were observed daily for any changes in their health. The use of other ectoparasiticides on dogs or in the environment was not allowed throughout the study period. However, individual treatments were authorized when heavy tick or flea infestations occurred. Due to the nature of the investigational veterinary product (collar), blinding was applicable only for the personnel that performed laboratory analyses.

**Table 1:** Study calendar and samples collected at the scheduled visits from screened/included dogs

**Study day**	**Date**	**Physical examination**	**Collar**	**Blood**	**Skin**	**Bone marrow**
Preliminary Screening	March-April 2012	+	-	+	-	-
Inclusion (D0)	April-May 2012	+	Application	+	+	+
I Follow-up (D90 ± 10)	July-August 2012	+	-	+	-	-
II Follow-up (D180 ± 10)	October-November 2012	+	-	+	-	-
III Follow-up (D210 ± 10)	November-December 2012	+	Removal	+	+	-
IV Follow-up (D300 ± 10)	February-March 2013	+	-	+	+	+

### Sampling and diagnostic procedures

Blood samples of approximately 5 mL were collected in vacuum tubes from the brachial or jugular vein, immediately transferred into cooled storage (+4°C), left to clot, and centrifuged (1,700 rpm for 10 minutes). The resulting serum was collected in individual microtubes. Skin tissue samples (about 0.5 cm^2^) were collected from the interscapular region and stored in individual microtubes containing 1 mL of phosphate buffer saline (PBS) solution. Bone marrow was sampled by an aspiration technique from the iliac crest using Rosenthal needles (16 or 18 gauge) and stored in the same way as blood and skin samples. In addition, bone marrow samples were smeared on slides for cytological examination for *L. infantum* amastigotes. Microtubes containing sera, skin and bone marrow samples were stored at -20°C until analysis.

Serum samples were tested for circulating anti-*L. infantum* antibodies by IFAT using a cut-off of 1:80 as described elsewhere [23]. Positive sera were also titrated using serial dilutions until negative. In addition, in order to reduce the risk of enrolling potentially infected dogs, serum samples collected at the preliminary screening were tested using 1:40 as cut-off. PCR for the amplification of *Leishmania* DNA was performed on bone marrow and skin samples. Total DNA was extracted using the QIAampDNA Micro Kit (Qiagen, GmbH, Hilden, Germany) and the Genomic DNA Purification Kit (Gentra Systems, Minnesota, USA), respectively, and a fragment of *L. infantum* kinetoplast DNA minicircle was amplified using the MC1/MC2 primer set [33]. Amplicons were resolved in ethidium bromide-stained (2%) agarose gels (Gellyphor, Italy) and sized by comparison with markers in the Gene Ruler™ 100 bp DNA Ladder (MBI Fermentas, Lithuania). Gels were photographed using a digital documentation system (Gel Doc 2000, BioRad, UK). Bone marrow smears were stained with MGG Quick Stain (Bio-Optica, Italy) and microscopically examined for *L. infantum* amastigotes. Each smear was examined for about 10 minutes under light microscopy (100 microscopic fields) using a 100× oil immersion objective.

### Entomological survey of sand fly activity

Light traps were used to assess the species and activity of sand flies in the investigated shelters. Starting from May 2012, two traps were placed biweekly in each shelter 50 cm above the ground before sunset (6:00 p.m.) and left *in situ* for 12 hours (6:00 a.m.) [32]. Monitoring activity was suspended after three consecutive negative trappings. Sand flies captured and grouped by shelter and collection day were counted and the species were identified using morphological keys [34,35].

### Statistical analysis

The minimum sample size per group (n = 107), was determined by Win-Episcope 2.0 [36] based on an expected incidence of 1% and 10% in G1 and G2, respectively (power = 90% and confidence level = 95%). The homogeneity of the groups was calculated retrospectively on day 0 baseline characteristics such as sex, age, coat length and body weight using a Chi-square test or Fisher’s exact test for qualitative data (sex, coat length) and analysis of variance (ANOVA) for quantitative data (age and weight). Since the objective of the study was to define the efficacy of the treatment in preventing *L. infantum* infection, negative dogs were defined as those that were negative in any of the diagnostic tests used (IFAT, PCR of skin and bone marrow tissues, cytology of bone marrow aspirate) throughout the study. The efficacy evaluation was based on the comparison of the incidence of *L. infantum*-infected dogs in the two treatment groups. Incidence was calculated as the annual crude incidence (i.e. considering only the results of the final sampling regardless of what happened in between) as follows: annual crude incidence = number of dogs newly infected with *L. infantum*/(number of negative dogs initially enrolled - number of lost or dead dogs) × 100. To overcome the problem of dogs lost to follow-up during the study, the incidence of infection was also calculated using the incidence density rate (IDR) [37]. IDRs were calculated at each follow-up as the number of newly infected dogs in any diagnostic test, divided by the number of dog-months of follow-up (i.e. the number of months between the previous and the current assessment for each dog at risk for *L. infantum* infection). Dogs tested only once (e.g. lost, dead) did not contribute at any time to the incidence calculation. Final IDRs were then expressed per year.

The efficacy (%) of the collar in preventing *L. infantum* infection was calculated using the formula: % protection = (% of positive dogs in group G2 - % of positive dogs in group G1)/(% of positive dogs in group G2) × 100. The percentage of positive dogs was calculated as the IDR. The significance of the efficacy (i.e. the significance of the difference between IDRs in collared and non-collared dogs) was tested using the Chi-square test.

## Results

At the preliminary screening 355 (78%) out of 455 dogs tested serologically negative for circulating anti-*L. infantum* antibodies (Table 2). The percentage of seropositive dogs was higher (*p* > 0.05) in S2 (25.4%; 79/311) than in S1 (14.6%; 21/144). On Day 0, 279 (G1 = 135, G2 = 144) dogs were enrolled; however, 60 dogs (G1 = 33, G2 = 27) were excluded from the study because they were either positive to serology and/or PCR on samples collected at day 0 (G1 = 26, G2 = 22), reluctant to keep the collar (G1 = 3), given for adoption (G2 = 1), dead before first follow-up visit (G1 = 2, G2 = 4) or because a history of leishmaniosis was reported (G1 = 2). Thus, a total of 219 (G1 = 102, G2 = 117) dogs were kept in the study and included in the calculation of efficacy. These dogs were homogeneously distributed (*p* > 0.05) with respect to sex, coat length, age and weight (Table 3). In the course of the study, 29 (G1 = 16, G2 = 13) were lost to follow-up at different time-points due to a fatal outcome of aggression (fights) (G1 = 11, G2 = 12), renal failure (G2 = 1), or they were excluded because they lost the collar more than 3 times (G1 = 5).

**Table 2:** **Results of screening for the presence of circulating anti-*Leishmania infantum *** **antibodies**

	**Study site**	
	**Messina (S1)**	**Augusta (S2)**
Number of screened animals	144	311
Mean age (months)/range	48.5/3-108	38.1/2-120
Coat length (%) short/medium/long	50.0/35.4/14.6	52.7/35.6/11.7
Sex (%) female/male	52.8/47.2	60.8/39.2
ELISA Positive	15/144 (10.4%)	33/311 (10.6%)
IFAT Positive	11/50 (22.0%)	69/268 (25.7%)
ELISA and/or IFAT positive	21/144 (14.6%)	79/311 (25.4%)

**Table 3:** Characteristics of dogs included in the study

	**All sites**			**Messina (S1)**		**Augusta (S2)**	
	**G1**	**G2**	**Total**	**G1**	**G2**	**G1**	**G2**
Number of included dogs	102	117	219	35	38	67	79
Mean age (months)/range	30.7/2-96	29.5/2-84	30.1/2-96	29.9/3-84	31.3/3-72	31.1/2-96	28.6/2-84
Sex (%) female/male	55.9/44.1	65.0/35.0	60.7/39.3	60.0/40.0	52.6/47.4	53.7/46.3	70.9/29.1
Weight (kg)/range	19.3/3.8-41	19.2/4.1-47	19.3/3.8-41	19.6/7.5-41	21.5/6.9-47	19.2/3.8-35	18.1/4.1-38
Coat length (%)							
Short	55.8	53.0	54.3	51.5	50.0	58.2	54.4
Medium	32.3	35.0	33.8	28.5	36.8	34.3	34.1
Long	11.9	12.0	11.9	20.0	13.2	7.5	11.5

In treated animals, collars were replaced if the dog’s weight increased above the 8 kg threshold (n = 8), or because they were damaged or lost on one (n = 10) or more (n = 3) occasions.

**Figure 2:**
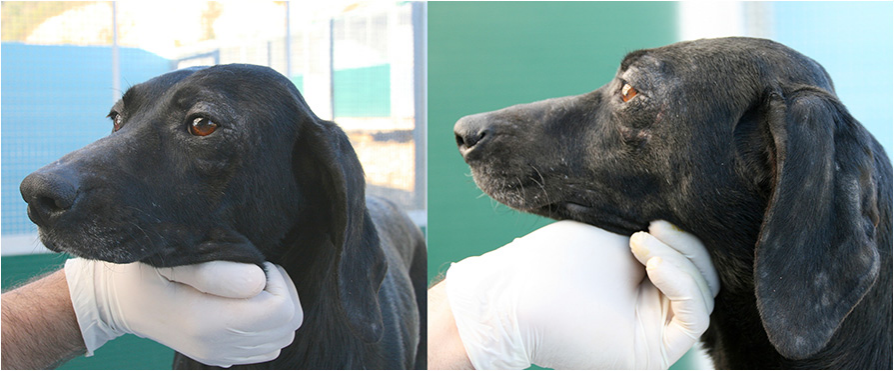
A control dog showing clinical signs of leishmaniosis (i.e. dry exfoliative dermatitis) at the last follow-up (D300).

Overall, three dogs (2.9%) in G1 and 47 (40.2%) in G2 tested positive for *L. infantum* in at least one of the diagnostic tests employed in the study. The number of seropositive dogs in G2 increased in the course of the study from 15 (first follow-up) to 41 (fourth follow-up), with some of them (n = 19; 40.4%) also positive in PCR on skin and/or bone marrow and/or in cytology on bone marrow aspirate (Table 4). All dogs except for one that had an initial seroconversion remained positive until the end of the study. Eight of the seropositive dogs (19.6%) in G2 showed an increase in antibody titers ranging from 1:160 to 1:1,280. Of the seropositive dogs in G1, two were found to be positive since the second follow-up, while the third tested serologically positive at the last follow-up (Table 4). At the last follow-up, some of the positive dogs in G2 displayed overt clinical signs suggestive of CanL, with lymph node enlargement (n = 28; 68.3%) and dry exfoliative dermatitis (n = 6; 14.6%) being the most frequent signs (Figure 2).

**Table 4:** **Results of diagnostic tests recorded in the follow-up of dogs in both groups (G1 = treated and G2 = control) that became positive for *Leishmania infantum *** **in at least one test/follow-up**

**Group**	**ID/Site**	**I Follow-up (July-Aug. 2012)**	**II Follow-up (October-Nov. 2012)**	**III Follow-up (November- Dec. 2012)**		**IV Follow-up (February-March 2013)**			
		**Serology**	**Serology**	**Serology**	**PCR skin**	**Serology**	**PCR skin**	**PCR bone marrow**	**Cytology**
G1	042/S2	negative	negative	negative	negative	1:80	negative	negative	negative
G1	261/S2	negative	1:80	1:160	negative	1:160	negative	negative	positive
G1	310/S2	negative	1:80	1:320	negative	1:320	negative	negative	positive
G2	073/S2	1:80	1:80	1:80	negative	1:80	negative	negative	negative
G2	081/S2	negative	negative	1:80	negative	1:80	positive	negative	positive
G2	083/S2	1:80	-	-	-	-	-	-	-
G2	086/S2	negative	1:80	1:80	negative	1:80	negative	positive	positive
G2	092/S2	negative	1:80	negative	negative	negative	negative	negative	negative
G2	093/S2	negative	negative	1:80	negative	1:80	negative	negative	positive
G2	106/S2	negative	negative	1:80	negative	1:80	negative	negative	positive
G2	109/S2	1:80	1:80	1:80	negative	1:80	positive	negative	negative
G2	111/S2	negative	negative	1:80	negative	1:80	negative	negative	positive
G2	112/S2	negative	negative	1:80	negative	1:80	positive	negative	positive
G2	140/S2	1:80	1:80	1:80	negative	1:80	positive	negative	positive
G2	142/S2	1:80	-	-	-	-	-	-	-
G2	157/S2	1:80	-	-	-	-	-	-	-
G2	158/S2	1:80	1:80	1:160	positive	1:1,280	positive	negative	negative
G2	163/S2	negative	negative	1:80	positive	1:160	positive	negative	negative
G2	164/S2	negative	negative	1:80	negative	1:80	negative	negative	negative
G2	168/S2	negative	negative	negative	negative	1:80	positive	negative	negative
G2	173/S2	1:80	1:80	1:80	negative	1:80	negative	negative	negative
G2	175/S2	negative	negative	1:80	negative	-	-	-	-
G2	177/S2	negative	negative	1:80	negative	1:80	negative	negative	negative
G2	178/S2	negative	negative	negative	negative	1:80	negative	negative	negative
G2	179/S2	negative	negative	1:80	negative	1:160	negative	negative	positive
G2	192/S2	negative	negative	1:80	negative	1:80	negative	negative	negative
G2	201/S2	negative	negative	1:80	positive	1:160	negative	positive	negative
G2	204/S2	1:80	1:80	1:80	negative	1:80	negative	negative	negative
G2	208/S2	1:80	1:80	1:80	negative	1:80	positive	negative	negative
G2	216/S2	1:80	1:80	1:80	negative	1:80	negative	negative	negative
G2	237/S2	negative	negative	1:80	negative	1:80	negative	negative	negative
G2	272/S2	1:80	1:80	1:160	negative	1:160	negative	negative	negative
G2	273/S2	1:80	1:80	1:160	negative	-	-	-	-
G2	277/S2	negative	negative	1:80	negative	1:80	negative	negative	negative
G2	298/S2	negative	negative	1:80	negative	1:80	negative	negative	negative
G2	018/S1	negative	negative	1:80	negative	1:80	negative	negative	negative
G2	021/S1	negative	negative	1:80	negative	1:80	negative	negative	negative
G2	022/S1	negative	negative	1:160	positive	1:160	negative	negative	negative
G2	051/S1	negative	negative	1:80	negative	1:80	negative	negative	negative
G2	062/S1	negative	negative	negative	negative	1:80	negative	negative	negative
G2	063/S1	negative	negative	1:80	negative	1:80	negative	negative	negative
G2	064/S1	negative	negative	1:80	negative	1:80	negative	negative	negative
G2	065/S1	negative	negative	1:80	negative	1:80	negative	negative	positive
G2	087/S1	1:80	1:80	1:80	negative	1:80	negative	negative	positive
G2	100/S1	negative	negative	1:80	negative	1:80	negative	negative	negative
G2	101/S1	negative	negative	negative	negative	1:80	negative	negative	negative
G2	139/S1	1:80	1:160	1:320	negative	1:320	negative	negative	negative
G2	140/S1	negative	negative	negative	negative	1:80	negative	negative	negative
G2	143/S1	negative	negative	1:160	positive	1:320	positive	negative	negative
G2	144/S1	negative	negative	1:80	negative	1:80	positive	negative	negative

For serology (IFAT) the highest dilution titer is reported.

The annual crude incidence calculated using dogs that remained in the study until the last scheduled follow-up (D300) was 3.5% (3/86) and 39.4% (41/104) for G1 and G2, respectively (*p* < 0.001). The IDRs for each group at each follow-up are shown in Table 5. The mean IDR at the final follow-up was 4.0% for G1 and 60.7% for G2. Hence, the mean efficacy of the collar in protecting dogs from *L. infantum* infection was 93.4% (*p* < 0.01), ranging from 90.5% to 100% at site S2 and S1, respectively. No adverse events or side effects related to the collars were observed in treated dogs. Severe tick infestations were recorded in 38 dogs in G2; these animals were individually treated by manual removal (n = 17) or with non-repellent individual ectoparasiticide product, i.e. Frontline^®^ spot-on, Merial SAS (n = 21).

**Table 5:** Incidence density rate for leishmaniosis in collared (G1) and uncollared control (G2) dogs calculated for each follow-up in the study

**Study day**	**Number of dogs in the cohort**		**Number of new cases**		**Dog-months of follow-up**		**Incidence density rate/year**	
	**G1**	**G2**	**G1**	**G2**	**G1**	**G2**	**G1**	**G2**
	**(Site S1/Site S2)**	**(Site S1/Site S2)**	**(Site S1/Site S2)**	**(Site S1/Site S2)**	**(Site S1/Site S2)**	**(Site S1/Site S2)**	**(Site S1/Site S2)**	**(Site S1/Site S2)**
Inclusion (D0)	102 (35/67)	117 (38/79)	-	-	-	-	-	-
I Follow-up (D90 ± 10)	102 (35/67)	117 (38/79)	0 (0/0)	15 (2/13)	299.9 (97.7/202.3)	341.6 (103.4/238.6)	0.0 (0.0/0.0)	52.7 (23.2/65.4)
II Follow-up (D180 ± 10)	91 (30/61)	100 (36/64)	2 (0/2)	2 (0/2)	273.9 (91.8/182.4)	308.0 (115.9/192.0)	8.8 (0.0/13.2)	7.8 (0.0/12.6)
III Follow-up (D210 ± 10)	86 (30/56)	95 (36/59)	0 (0/0)	25 (10/15)	81.7 (29.1/52.1)	89.30 (34.9/55.5)	0.0 (0.0/0.0)	336.0 (343.6/324.6)
IV Follow-up (D300 ± 10)	84 (30/54)	67 (26/41)	1 (0/1)	5 (3/2)	242.8 (87.0/155.5)	190.3 (75.4/115.2)	4.9 (0.0/7.7)	31.5 (47.8/20.8)
Total			3	47 (15/32)	898.3 (305.6/592.3)	929.2 (329.6/601.3)	4.0 (0.0/6.1)	60.7 (54.6/63.9)

Site S1 = Messina shelter; Site S2 = Augusta shelter.

Sand flies (n = 700) were captured from the end of May 2012 until the first week of November 2012 (S1), and from the end of June until October (S2). At both sites *Sergentomyia minuta* was the best-represented species (433 females and 83 males; 73.7%), followed by *Phlebotomus perniciosus* (84 males and 35 females; 16.9%), *Phlebotomus perfiliewi* (42 females and 13 males; 7.9%) and *Phlebotomus neglectus* (7 females and 2 males; 1.3%). A single female specimen of *Phlebotomus sergenti* was captured at site S2. The largest number (n = 327) of sand flies was captured in September, while the majority of *P. perniciosus* specimens were collected in August (n = 21), September (n = 40) and October (n = 25), respectively.

## Discussion

The slow-release collar tested in this study proved to be safe and highly effective (mean efficacy at the final follow-up = 93.4%) in preventing *L. infantum* infection in dogs living in an area hyper-endemic for CanL. A single preliminary field trial suggested the high potential of this collar in preventing CanL in young dogs never exposed to a transmission season [30]. In the present study we confirmed and extended this knowledge by testing the collar in a larger and heterogeneous dog population from an area hyper-endemic for CanL. The hyperendemicity of both study sites was confirmed by the annual incidence (39.4%) and the mean IDR (60.7%) recorded in the control dogs at the last follow-up, which are the highest ever recorded in southern Italy [19-21,23,30,38-40]. This might be due to the simultaneous presence of diseased animals and susceptible unprotected dogs in the two shelters, along with the presence of competent sand fly species. In addition, according to the results of the preliminary screening, a large number of apparently healthy but seropositive dogs were present at both study sites (S1 = 14.6%; 21/144 and S2 = 25.4%; 79/311).

In the course of the study, a total of 47 (40.2%) out of the 117 uncollared dogs tested positive at least in one of the four follow-up investigations. The majority of these dogs were positive only on serology while others (n = 19/47; 40.4%) displayed an active infection, being simultaneously positive by different diagnostic methods (serology, PCR and cytology). The status of active infection was also corroborated by the increase in the antibody titers recorded in eight dogs (19.6%). It is known that, once established in dogs, *L. infantum* infection progresses over a variable period of time through different clinical presentations [7,38,40], although in the 12 months post infection the majority of dogs do not display a clinical condition [40]. Accordingly, in the present study, while lymph-node enlargement was the clinical sign most frequently found (68.3%) in serologically positive dogs, other signs such as dry exfoliative dermatitis and weight loss were observed at the last follow-up in 14.6% and 2.4% of dogs, respectively.

Among the collared dogs, only three animals became seropositive for *L. infantum*. Although seropositive, these animals did not show clinical signs suggestive of CanL. These three dogs were housed at site S2, where the highest IDR of the infection (63.9%) in control dogs was registered at the last follow-up. This might explain the high parasitic pressure at site S2, which ultimately led to a percentage of protection below 100%. It is accepted that the mechanism through which the collar protects dogs against *Leishmania* infection is the anti-feeding effect of flumethrin that reduces the number of sand flies bites and, in turn, the infection challenge [30]. In the same way, it has been demonstrated that a small number of sand flies may effectively feed on collared dogs, as demonstrated in a study with dogs wearing deltamethrin-impregnated collars [41]. Nonetheless, most sand flies fed on collared dogs will die in a few hours [41]. Thus, it may be concluded that even if some collared dogs may become infected if bitten by an infected sand fly, they will hardly ever contribute to transmission of the infection to other dogs. Indeed, most sand flies will be repelled when in contact with a collared dog and the few that will be able to feed on them will die shortly afterwards.

The percentage efficacy of the collar containing imidacloprid (10%) and flumethrin (4.5%) against *L. infantum* infection recorded in young dogs (100%) [30] or in the present study (93.4%) is higher than that obtained using a deltamethrin-impregnated collar, which yielded a maximum 86% after two consecutive seasons in southern Italy [19,20]. This might be due to the longer residual effect of the imidacloprid/flumethrin collar (up to 8 months) than the deltamethrin collar (up to 5 months) as a result of the slow-release mechanism of the first device. In the present study, treated dogs wore the collar for up to eight months (from May to December). Accordingly, sand fly species that are competent vectors of *L. infantum* (*P. perniciosus*, *P. perfiliewi* and *P. neglectus*) were captured from June to October and from May to November at sites S1 and S2, respectively. The retrieval of a single specimen of *P. sergenti* in S2 is also of interest considering that this species is involved in the transmission of *Leishmania tropica*, the aetiological agent of human cutaneous leishmaniosis in the Middle East and Africa [2].

Despite the protracted period for which the collars were worn and the typology of the treated animals, i.e. sheltered dogs living in collective pens separated by iron wire meshes, no side effects or accidents were recorded during the study. The percentage (22.5%) of collar replacements observed in this study is similar to that reported in a previous investigation involving the same collar [30]. In fact, the dogs that had lost the collar were mainly young, probably very playful and lively animals.

## Conclusions

The slow-release collar containing a combination of 10% imidacloprid and 4.5% flumethrin tested in this study proved to be safe and highly effective (90.5–100%) in preventing *L. infantum* infection in a large and heterogeneous group of dogs. The labeled protection conferred by a single collar (eight months) spanned an entire sand fly season in the examined area, where competent sand flies are active from May to November. Therefore, the regular use of collars, at least during the sand fly season, may be regarded as a reliable and sustainable strategy for the control of leishmaniosis in dogs living in or travelling to an area endemic for CanL.

## Competing interests

DS is an employee of Bayer Animal Health GmbH, Leverkusen, Germany. ARCOBLU S.R.L. is an independent Contract Research Organization, which was contracted to manage and monitor this study. FSB is the project manager of ARCOBLU S.R.L. All authors voluntarily publish this article and have no personal interest in this trial other than publishing the scientific findings.

## Authors’ contributions

EB, DO, FDT, DS and FSB conceived and designed the survey. EB, GG, EN, LF, CP, VDT, MSL carried out the field survey and laboratory work. GG performed the entomological study. FSB monitored the study. FSB and GC performed the statistical analysis of data. EB and DO drafted the first version of the manuscript. All authors have contributed to the revision of the manuscript and approved its final version.
